# An interview with Irismar Reis de Oliveira, Section Editor for Clinical Psychology and Psychotherapy

**DOI:** 10.1186/2050-7283-1-4

**Published:** 2013-02-27

**Authors:** Irismar Reis de Oliveira

**Affiliations:** Departamento de Neurociências e Saúde Mental, Universidade Federal da Bahia, Salvador, Brazil

## How did you first become interested in psychotherapy?

My decision to become a psychiatrist and psychoanalyst dates back to the early 1970s, a few years before I entered medical school at the age of 21. At that time, I had read all the books written by psychoanalysts Karen Horney and Erich Fromm. However, my interest in psychological literature had started during adolescence, when I was lucky enough to be introduced to Arthur Harry Chapman, an American psychiatrist who used to write psychotherapy books, and who decided to migrate to my hometown, Vitória da Conquista, in Brazil. Thus, just out of adolescence, I had the privilege of discussing with a renowned American scholar about my intention of enrolling in medical school and becoming a psychiatrist and psychotherapist. His work (e.g., [1, 2]) relied clearly on the ideas of Harry Stack Sullivan, the founder of the interpersonal movement in psychotherapy. Also importantly, I was exposed to autogenic training–a relaxation technique developed by the German psychiatrist Johannes Schultz–as a client in the late 1960s, when I was still a teenager.

However, during my medical course, I lost interest in psychiatry and, at its conclusion, I applied for a residency in cardiology, after which I had a 2-year clinical practice working in this specialty. At that time, I also joined the Department of Pharmacology at the Federal University of Bahia, in Salvador, Brazil, as an assistant professor. Unfortunately (today I would say fortunately), cardiology did not fulfill my professional and personal expectations, and the original idea of becoming a psychoanalyst came to mind again. So, I registered at a psychoanalysis-training institute and started personal analysis, having read most of Freud’s books at that time. My psychoanalysis training would continue in 1983, in Paris, where I completed a 4-year residency in psychiatry. Initially and simultaneously with my psychiatric training at Sainte-Anne Hospital in Paris, I resumed personal analysis and followed a psychoanalysis university course during which I was trained in Lacanian psychoanalysis for 2 years. So, both personal analysis and psychoanalysis training in Brazil and in France summed up 5 years, after which, still living in Paris, I decided to focus on clinical psychiatry and psychopharmacology. In 1987, I received certification as a specialist in psychiatry from the Faculté de Médicine Cochin Port Royal, University of Paris V.

Returning to Brazil in 1988, I resumed teaching and started research activities at the Department of Pharmacology, at Federal University of Bahia, where I would remain until 1996. In 1995, I had received a Ph.D. in neuropsychopharmacology, and in 1996 I joined the Department of Neurosciences and Mental Health at the same university, as an associate professor. In 1998, I rediscovered psychotherapy and registered for the first extramural cognitive therapy course by the Beck Institute for Cognitive Therapy and Research (now the Beck Institute for Cognitive Behavior Therapy) being held in Brazil.

The year 2000 was a turning point in my professional life because I: (1) received a certification in cognitive therapy from the Beck Institute, in Philadelphia; (2) joined the Academy of Cognitive Therapy (http://www.academyofct.org) as a founding fellow; (3) became full professor of psychiatry at the Department of Neurosciences and Mental Health, Federal University of Bahia; and (4) became head of the psychiatry service at the university hospital. I was gradually shifting clinical practice, teaching, and research interests from an exclusive biological and psychopharmacological focus to an integrative focus linking psychopharmacology and psychotherapy [3]. A multicenter randomized clinical trial (RCT) of topiramate and cognitive-behavior therapy (CBT) in the treatment of binge-eating disorder was the first result of this shift [4]. I did not realize at the time that I was also going in the direction of the already existing psychotherapy integration movement.

## Why is it an exciting time to be involved in psychotherapy in particular?

In about a century and a half, since Freud developed psychoanalysis, there has been an explosion of different modalities of psychotherapies, enumerated as 551 by Kazdin [5], and this number is expanding. Despite this large number, and perhaps as a reaction to the long-lasting hegemony of psychoanalysis, there was a decline in the attention given to psychotherapy, at least by psychiatrists, and a great increase of psychopharmacology dominance was observed, stimulated by the discoveries in biological psychiatry and psychopharmacology, as well as the influence of the pharmaceutical industry. Unfortunately, however, although safer than the older drugs, the new drugs put onto the market since the 1990s did not completely fulfill the initial promises, and psychiatric drug treatment remained an unmet need. Furthermore, there seems to be a plateau or even a decrease in the development of new drugs in the last few years, and this might be a possible explanation for the rediscovery of the power of psychotherapy [6]. This rediscovery has also been supported by the growing number of well-designed studies not only showing that psychotherapy works, but that it works as well as, and sometimes to a greater degree and longer, than drugs in certain clinically relevant psychiatric conditions, like depression, anxiety, marital dissatisfaction, substance abuse, health problems and sexual dysfunction, as demonstrated by numerous RCTs [7]. It also seems to be more enduring both in clinical trials and in real world studies [8].

## What does the future hold for psychotherapy research and what do you think are its limitations?

Referring to the future of psychotherapy research calls for some reflections on its past and present. Historically, psychoanalysis was the “first force,” and was followed by behaviorism (second force) as a reaction to the “unscientific” nature of psychoanalysis theory and lack of connection to observable phenomena. Some prominent behaviorists, however, would later expand their models to incorporate cognitions, as proposed by Albert Ellis, Aaron Beck and others, and which emphasized the importance of mental representation of the stimuli as central elements to psychopathology. The third force emerged after World War II and was referred to as the humanistic and experiential approaches because these theoretical approaches were based on the humanistic philosophers. Humanistic and experiential treatments were mainly represented by person-centered therapy developed by Carl Rogers, and Gestalt therapy developed by Frederick Perls. There have also been the fourth (feminist and multicultural theories) and the fifth (post-modern and constructivist theories) forces [8].

Importantly, the approaches encompassed by each force continued to evolve, resulting in multiple spinoffs. Thus, it is not surprising that the huge number of approaches originated from the above-mentioned forces in psychotherapy called for the need for assessing the efficacy of such treatments, and gave rise to the “empirically validated treatments” project [9], which reduced the number of validated psychotherapies to the fewer than 50 which demonstrated superiority over some type of placebo by at least two RCTs. The word “validated” was later replaced with “supported,” becoming “empirically supported treatments” (EST).

Another movement derived from medicine, namely evidence-based practice (EBP), was adopted by psychologists (APA Presidential Task Force on Evidence-Based Practice, [10]) who defined EBP as “the integration of the best available research with clinical expertise in the context of patient characteristics, culture, and preferences,” and endorsed the multiple types of evidence that contribute to effective practice in psychology, such as efficacy, effectiveness, cost-effectiveness, cost-benefit, epidemiology and treatment utilization [8].

Another consequence of the huge number of single-school psychotherapy approaches was a movement that started in the 1970s and gave rise to what came to be known as the psychotherapy integration movement, which encompassed common factors (an aspect of psychotherapy that is present in most, if not all, approaches to treatment), theoretical integration (synthesis of two or more theories into a single conceptualization), technical integration (use of techniques drawn from several different therapeutic approaches), and assimilative integration (one theoretical position is accompanied by a willingness to incorporate techniques from other therapeutic approaches) [11, 12].

Thus, my understanding is that what the future holds for psychotherapy research is the challenge of answering or clarifying many questions still incompletely explored, some of which are: How does psychotherapy work? Are some psychotherapies more effective than others? What is the role of theory in psychotherapy? Is psychotherapy integration a solution for the complexities of the field? Some of these questions are approached in the books of the *Theories of Psychotherapy Series*, organized by Carlson and Englar-Carlson [12]. For those interested in knowing more, I suggest you read *The basics of psychotherapy: An introduction to theory and practice*[8], followed by *Psychotherapy integration*[11], before going to each individual psychotherapy theory.

Limitations to psychotherapy integration, in my opinion, may be more in excessive reliance of psychotherapists on their theoretical allegiances and their unwillingness to talk to those from other theories than those from inside the field itself.

## What challenges and developments can we expect to see in the next few years in psychotherapy, and how can research contribute to meeting these challenges?

I will approach this topic citing my own research, Trial-Based Therapy (TBT) [6], as an example of assimilative psychotherapy integration [13]. In this kind of integration, various techniques from different theoretical origins are incorporated within the context of understanding provided by the home theoretical approach [11]. TBT relies on CBT as the organizing theory and adds technical interventions drawn from several other approaches. Among them are gestalt, experiential, acceptance and commitment therapy, compassion-focused therapy, metacognitive therapy, mindfulness, and Mitchell’s [14] two-person relational model. Furthermore, by adding literature, TBT relies on the work of Franz Kafka, *The Trial*, and some of its techniques incorporate a law metaphor, by which the patient expresses multiple internal characters (e.g., prosecutor, defense attorney, witnesses, jurors, etc.) to challenge his/her core beliefs conceptualized as self-accusations [15]. As TBT is a 3-level, 3-phase, structured step-by-step approach, and its conceptualization involves a cyclic interactional mechanism in which each component in each level influences the other, this approach is flexible enough to allow the therapist to adapt the treatment to the individual’s needs and characteristics.

## What topics are currently trending within the field of psychotherapy?

Stahl’s [16] conceptualization of psychotherapies as epigenetic “drugs” is very attractive. It further strengthens the idea that psychotherapy and drugs have a common underlying mechanism of change. According to Stahl, learning and environmental experiences, such as psychotherapy, change brain circuits, as do drugs. Thus, both psychotherapy and psychopharmacology can be clinically effective for treating psychiatric disorders, and their combination can be therapeutically synergistic. Psychotherapy and many other forms of learning might induce epigenetic changes in brain circuits that would enhance the efficiency of information processing in malfunctioning neurons to improve symptoms in psychiatric disorders, just like drugs. Epigenetics might be a trend in psychotherapy research.

Another trend in psychotherapy research is further evaluating the efficacy, as well as consolidating the new generation of psychotherapy approaches, both in the psychodynamic and CBT umbrellas, relative to the more traditional approaches. Examples of new psychodynamic approaches are short-term anxiety-regulating psychotherapy [17], brief relational therapy [18] and time-limited dynamic psychotherapy [19], and examples of CBT approaches are acceptance and commitment therapy [20], dialectical behavioral therapy [21], metacognitive therapy [22], mindfulness-based cognitive therapy [23] and compassion-focused therapy [24].

## Are there any particular papers you would like to see submitted to your section?

Papers addressing psychotherapy integration, psychotherapy-psychopharmacology integration, and mechanisms of change in psychotherapy might be interesting ones.

## What advantage do you think BMC Psychology has over other journals?

First and foremost, BMC is a highly regarded editorial group, publishes multiple journals in many different areas, and as such, will make BMC Psychology one of the most important sources of high quality research in a broad range of disciplines in psychology. This and being an open access journal make it a very advantageous source of publication in psychology.

## Do you think psychotherapy would benefit from an open access journal, such as BMC Psychology?

Open access to knowledge is a trend in science with which I agree and adhere to, as demonstrated by my open access cognitive therapy book recently published [25]. As far as I know, there are few open access psychology journals relative to other fields.

## As you know BMC Psychology will operate an open peer review process. How important do you think this is to the field?

Peer review is the only way to assure quality and credibility of publications. However, Mahoney [26] called attention to confirmatory bias, defined as the tendency to emphasize and believe experiences that support one’s views and to ignore or discount those which do not. After interviewing 75 reviewers to referee manuscripts which described identical experimental procedures, but which reported positive, negative, mixed, or no results, it was concluded that reviewers were strongly biased against manuscripts reporting results contrary to their theoretical perspective. BMC Psychology is committed to an open and integrative work, as demonstrated by the distinct orientations of the section and associated editors list.

## What made you interested in joining BMC Psychology?

Although I am a psychiatrist, and have been publishing mainly in psychopharmacology and biological psychiatry, I also have been teaching psychotherapy for at least 10 years. More than 90% of my psychotherapy workshops attendants in Brazil and in Europe are psychologists (contrasting with the USA, whose attendants are predominantly psychiatrists). Joining BMC Psychology as a psychotherapy section editor presented to me as a great opportunity to expand communication with scholars in the field of psychotherapy all over the world.

## Author’s information

Irismar Reis de Oliveira, MD, PhD, is a Professor of Psychiatry in the Department of Neurosciences and Mental Health, Federal University of Bahia, Brazil. He is the Section Editor for the Clinical Psychology and Psychotherapy section for *BMC Psychology*. In this interview we find out a little more about Professor Reis de Oliveira and the key issues in the field of psychotherapy research.
